# Analysis of the Overlength Main Noncoding Region in *Metacarcinus magister* (Decapoda: Brachyura) and a Phylogenetic Study of the Cancroidea Species

**DOI:** 10.3390/genes15040437

**Published:** 2024-03-29

**Authors:** Zhengfei Wang, Xinyi Xu, Yuqing Zheng, Zhiwen Xu, Yunjie Li, Huohuo Chen

**Affiliations:** Jiangsu Key Laboratory for Bioresources of Saline Soils, Jiangsu Synthetic Innovation Center for Coastal Bio-Agriculture, Jiangsu Provincial Key Laboratory of Coastal Wetland Bioresources and Environmental Protection, School of Wetlands, Yancheng Teachers University, Yancheng 224001, China; 18082165776@163.com (X.X.); 19895621808@163.com (Y.Z.); 15722573182@163.com (Z.X.); 19825989363@163.com (Y.L.); 17715213178@163.com (H.C.)

**Keywords:** mitogenome, Brachyura, Cancroidea, noncoding region, phylogeny

## Abstract

Complete mitochondrial genomes (mitogenomes) can provide important information regarding the molecular evolution and phylogenetic relationships of marine invertebrates, especially in Brachyura. Only one Cancroidea species of mitogenomes has been sequenced before; in this research, the mitogenomic characteristics of *Metacarcinus magister* (Cancridae: Cancroidea) are newly studied. The length of the *M. magister* mitogenome was 48,820 bp, and it contained the typical 13 protein-coding genes, 2 ribosomal RNA genes, and 22 transfer RNA genes. We performed a series of analyses on the characteristics of the mNCR of *M. magister*. The phylogenetics, life circumstances, and selective pressures were all analyzed to explain the formation of this length, which revealed the length of the *M. magister* mitogenome to be approximately three times greater than the normal length of Brachyuran mitogenomes. Phylogenetic analyses based on a dataset of 215 Decapodan mitogenomes indicated that all Eriphioidea crabs were clustered together as a group. Moreover, the rearrangement mechanism of the Cancroidea species was predicted to provide stronger evidence for the phylogenetic analysis. In general, the results obtained in this study will contribute to a better understanding of the cause of the unusual length of the *M. magister* mitogenome and provide new insights into the phylogeny of Brachyura.

## 1. Introduction

In recent years, analyses of molecular data have played an important role in research on the deep phylogenetic relationships of marine invertebrates, and they have especially reshaped our understanding of the evolutionary relationships of marine crabs [1]. Since a great deal of biological information is contained in mitochondrial genomes, comparative studies on marine animals’ mitochondrial genomes have received more and more attention in recent years, whether they are concerned with such animals’ morphology or genetics [2]. The mitogenomes of metazoan crabs are generally in the range of 14–19 kilobases (Kb) in length, and a typical circular molecule contains 37 genes and 13 mitochondrial-encoded proteins (PCGs), i.e., ATPase subunits 6 and 8 (*atp6* and *atp8*), cytochrome oxidase (subunits 1–3 (*cox1–3*), cytochrome b (*cob*)), dehydrogenase subunits 1–6, and 4L (*nad1–6* and *nad4L*). These PCGs play important roles in the electron transport chain, which produces nearly 95% of adenosine triphosphate (ATP) for essential energy production [3]. Two ribosomal RNAs (rRNAs), L and S (*rrnL* and *rrnS*), 22 tRNAs, and one putative main noncoding region (mNCR) are also present [4,5]. Compared with signal-based molecular markers, complete mitogenomes contain more biological information and can allow the interference of nuclear pseudogenes to be effectively avoided. Moreover, based on a variety of their characteristics, such as their haploid nature, limited recombination, material inheritance, and rapid mutation rate, mitogenomes have been widely used in evolutionary and population studies [6,7,8,9]. In marine species, a complete mitogenome can provide various evolutionary traits that have been widely investigated, including phylogenetics, population genetics, and molecular evolution [10,11].

Brachyura (true crabs), the crustacean decapod infraorder with an early to mid-Jurassic origin, is one of the most species-rich and highly developed groups of extant crustaceans [12,13], and it includes more than 7250 known species in 104 families. As the most diversified and advanced aquatic invertebrates, today, these crabs are distributed worldwide [14,15]. Brachyura species have been found in a variety of environments, including brackish and fresh waters, inland from the ocean, and even in some harsh habitats, such as hydrothermal seep [16]. The majority of the compelling evidence for the evolutionary relationships of Brachyura originates from morphological studies [17]; today, phylogenetic research is continued through various molecular studies based on partial mtDNA sequences [18]. As an epibenthic marine invertebrate, *M. magister* is principally distributed throughout Southeast Asia and in estuaries and nearshore environments along the west coast of the American continents, from Alaska to Baja, on sedentary or sandy seafloors with a depth of 30–100 m. These complicated habitats, such as oyster beds, eelgrass, and oyster reefs, can provide the perfect protection and food for *M. magister*. In recent years, most research surrounding *M. magister* has concentrated on its morphology, economy, and aquaculture; however, *M. magister* and Cancridae have rarely been researched at the molecular level. 

This study makes a thorough inquiry into the evolution of *M. magister* based on its complete mitochondrial genome. We characterized mitochondrial gene orders, nucleotide compositions, and gene contents. In particular, a special ultra-long mNCR (main noncoding region) was found in *M. magister*, which was different from that in other Brachyura crabs. Moreover, the available complete mitogenome sequences were used to support phylogenetic and gene rearrangement analyses of Cancroidea.

## 2. Materials and Methods

### 2.1. DNA Extraction

Two samples of *M. magister* were taken from Baoshan District, Shanghai, China, on the date of 20 August 2018 (Figure S1). One of the samples was stored as a copy in a freezer at −80 degrees at Yancheng Teachers’ University, and the specimen catalog number was 082003. The other one was dissected for muscle samples, which were flash-frozen in liquid nitrogen and stored in a freezer at −80 degrees to extract the total genomic DNA. The muscle tissues of the samples were used to extract genomic DNA with the Aidlab Genomic DNA Extraction Kit, and the specific steps were performed as per the instructions provided by the manufacturer (Aidlab Biotech, Beijing, China). 

### 2.2. PCR Amplification and Sequencing

The mitogenome of *M. magister* was sequenced with next-generation sequencing (Illumina HisDeq 4000; Shanghai Origingene Bio-pharm Technology Co., Ltd., Shanghai, China), and the raw sequence data of the species used in this research were deposited in the Short Read Archive (SRA) database (https://www.ncbi.nlm.nih.gov/sra, (accessed on 1 August 2022)). In order to determine the accuracy of the species samples, a 25 μL mixed system was used, and PCR was run on an ABI9700 DNA amplification apparatus with the universal primer *cox1* and *16SrRNA* (Table S1). The reaction system was as follows: 12.5 μL of 2-X F8 PCR MasterMix, 1 μL of DNA template, 10.5 μL of ddH_2_O, 0.5 μL of reverse primer, and 0.5 μL of forward primer. The PCR amplification conditions were as follows: The templates were pre-denatured at 95 °C for 4 min (initial denaturation); then, the denaturation lasted for 30 s when the temperature was changed to 94 °C (denaturation). The third step was renaturing at 55 °C for 30 s (annealing) and 72 °C for 30 s, followed by a 10 min extension at 72 °C. The thermocycling profile was operated in 34 cycles. The results demonstrated that the sequences were identical to those obtained through Illumina sequencing. 

### 2.3. Sequence Analysis and Gene Annotation

The mitogenome sequences were searched using BLAST (http://blast.ncbi.nlm.nih.gov/Blast, (accessed on 1 August 2022)) on the NCBI website and compared with the published complete mitogenome of *Cancer pagurus* in order to ensure that the sequences were correct [19]. The complete mitogenome was uploaded to GenBank (accession number OR400215, available at NCBI). By using ORF Finder via NCBI and the MITOS Web Server (http://mitos2.bioinf.uni-leipzig.de/index.py, (accessed on 1 August 2022)), the PCGs were determined with the invertebrate mitochondrial genetic code that was selected [20]. Moreover, the MITOS Web Server and tRNAscan-SE (http://lowelab.ucsc.edu/tRNAscan-SE/, (accessed on 1 August 2022)) were used to calculate the potential stem-loop secondary structures within the tRNA sequences [21]. Start codons and stop codons in *M. magister* that were abnormal in comparison with those of other crabs were identified, and relative synonymous codon usage (RSCU) was performed with MEGA 5; this was calculated by using PCGs without incomplete codons [22]. All of the analyses of compositional skews were conducted with the following formulas: AT-skew = (A − T)/(A + T) and GC-skew = (G − C)/(G + C). Gene maps of the *M. magister* mitogenomes were drawn with the online mitochondrial visualization tool Organellar Genome DRAW and BRIG [23,24]. The secondary structure of the mNCR was predicted with the UNAfold Web Server (http://www.unafold.org/mfold/applications/dna-folding-form.php, (accessed on 1 August 2022)) [25]. Because of the special length, DNAMAN was used in multiple-sequence alignment between the mitogenome of *M. magister* and the mNCR [26]. Tandem repeats of the mNCR were identified via the Tandem Repeats Finder Server (http://tandem.bu.edu/trf/trf.html, (accessed on 1 August 2022)) [27].

### 2.4. Phylogenetic Analysis

To reconstruct the phylogenetic relationships among Brachyura crabs, the newly sequenced crab mitogenomes, as well as 215 previously sequenced mitogenomes within Dacapoda, were used in phylogenetic analyses (Table S2). A total of 17 Anomura species were used as outgroup taxa. The nucleotide sequences of the 13 PCGs were concatenated for this research, and all of the data on the 215 species were downloaded from Genbank. The nucleotide sequences of the 13 PCGs were converted into amino acid sequences with MEGA 5. We aligned the amino acid sequences for each of the 13 protein-coding mitogenomes from 216 species by using MUSCLE 3.8 in MEGA 5 [28], and the final concatenated set of amino acid sequences was used for further phylogenic analyses. MtArt + I + G was chosen with jModeltest as the best substation model for our data. The phylogenic relationships of *M. magister* were estimated by reconstructing the phylogenetic tree, and the Bayesian inference (BI) and maximum likelihood (ML) methods were carried out with MrBayes v3.2.6 [29] and RaxML v8 [30]. The trees were visualized with the program IQ-TREE 1.5.2 [31]. For the analyses with BI, we conducted two simultaneous runs of 10,000,000 generations, which sampled trees every 1000 generations, with one cold chain to encourage swapping among the Markov chain Monte Carlo (MCMC) chains and three heated chains. Tracer 1.6 was used for the convergence of the sampled parameters and potential autocorrelation (effective sampling size for all parameters > 200) (http://tree.bio.ed.ac.uk/software/tracer/, (accessed on 1 August 2022)). Furthermore, the deviation of the average standard split frequencies between both runs was monitored in order to ensure that it remained less than 0.01. After discarding the first 25% of the trees as a “burn-in”, Bayesian posterior probabilities were required as a 50% majority rule from trees with sampling at stationarity. The ultimate phylogenetic trees were visualized in FigTree v1.4.2 [32].

### 2.5. Gene Rearrangements

When differences were discovered in annotations, mitogenomes were reannotated with MITOS and manually corrected to ensure the accuracy of the data. In order to infer putative ancestral gene orders and relationships between the Brachyuran crabs that were researched here, we applied the output of the previous step to software such as Common Interval Rearrangement Explorer (CREx) [33]. The CREx program is an important tool in the analysis of gene rearrangements (http://pacosy.informatik.uni-leipzig.de/crex, (accessed on 1 August 2022)), and pairwise comparisons were performed with the CREx program between different mitogenomes. This program was used to determine pairwise rearrangement events in genomes [33], and it mainly analyzed genomic rearrangement pathways using common intervals [34] (a common interval is a subset of genes that are contained in two or more investigated mitogenomes consecutively) [35]. CREx considers transpositions, inversions, inverse transpositions, and TDRLs (tandem duplication random loss), and it can heuristically identify the most parsimonious transformational pathways that connect one mitogenome to another mitogenome once the whole set of common intervals has been determined, and vice versa [35]. However, during the analysis, this program could only output a single transformation path; the current version did not explore all possible alternatives. Therefore, further deductions were made through the results of the analysis with CREx; the conclusions and some details will be explained in the sections presenting the results and discussion.

## 3. Results

### 3.1. Mitogenome Organization and Base Composition

**Figure 1:**
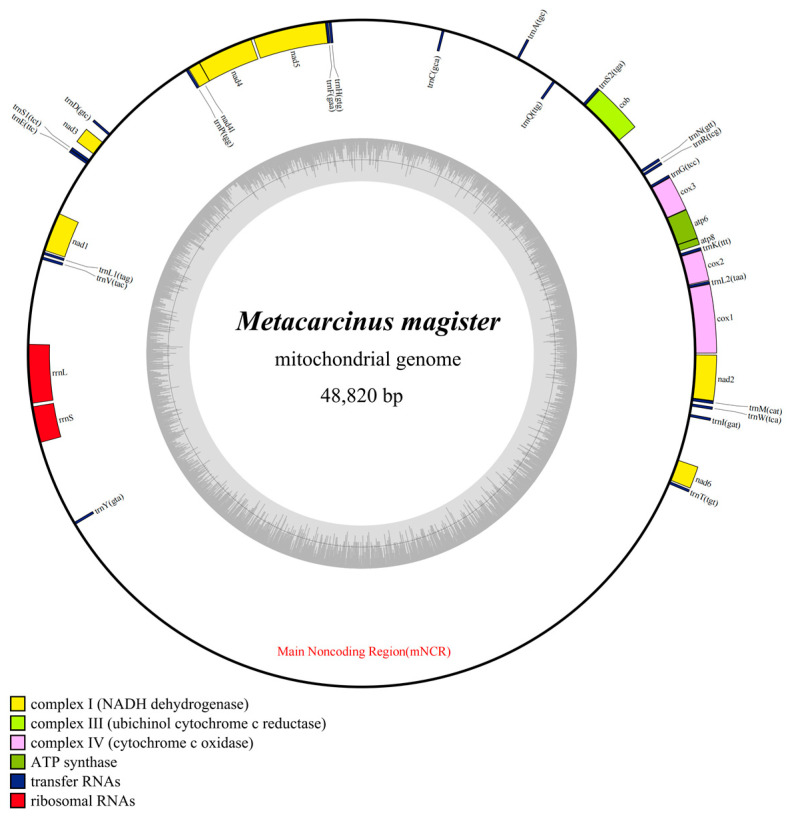
Mitochondrial genome maps of *M. magister*. Protein-coding genes are color-coded (cox: lavender; nad: yellow; atp: green; cob: kelly); rRNA genes are in red; tRNA genes are in blue. The abbreviations of protein-coding genes are the following: *atp6* and *atp8* for ATP synthase subunits 6 and 8, *cox1–3* for cytochrome oxidase subunits 1–3, cob for cytochrome b, *nad1–6* and *nad4l* for NADH dehydrogenase subunits 1–6 and 4L, *rrnL* and *rrnS* for large and small rRNA subunits, and mNCR for the main noncoding region.

The mitogenome of *M. magister* included 37 typical mitochondrial genes, the length of which was 48,820 bp. In addition, 23 genes (9 PCGs and 14 tRNAs) were encoded on the heavy strand (H-strand), and 14 genes (4 PCGs, 8 tRNAs, and 2 rRNAs) were encoded on the light strand (L-strand) (Figure 1). The longest gene of *M. magister* was *nad5* (1689 bp), and the lengths of the shortest four genes were equal (*trnL2*, *trnG*, *trnF,* and *trnD* with only 64 bp) (Table 1). The total overlapping length, which contained six overlapped regions (the parts shared between genes), was 51 bp, and the longest overlap region was 17 bp. One was located between *cob* and *trnS2*, and the other was located between *trnF* and *nad5*. A remarkable feature of the *M. magister* mitogenome was the presence of intergenic spacers (IGSs), which spanned 34,004 bp. IGSs existed between nearly all of the mitochondrial genes, i.e., in 29 out of the 37 possibilities, ranging from 6 bp to 17,097 bp. Six of them consisted of more than 1000 bp, and the longest one contained 17,097 bp and was located between *trnY* and *trnT*. 

### 3.2. Skewness

From the perspective of nucleotide composition, the entire mitogenome of *M. magister* exhibited AT bias (62.57–73.18%) (Table 2). The nucleotide composition of the *M. magister* mitogenome was biased as follows: A = 33.97%, T (U) = 34.72%, G = 8.97%, and C = 22.34%. The A + T content was 68.69%, and the G + C content was 31.31%; these proportions led to a subsequent bias in the homologously encoded amino acids. The AT-skew (−0.01) and GC-skew (−0.43) of the whole mitogenome were negative; these data were similar to those of most species in Brachyura [36]. The AT-skews of most Brachyuran mitogenomes are negative, meaning that Ts are more abundant than As. The GC-skews for all Brachyuran mitogenomes are negative, indicating the occurrence of more Cs than Gs.

### 3.3. PCGs and Codon Usage

The 13 PCGs of *M. magister* were similar in length and arrangement to those in other sequenced Brachyuran mitogenomes. The region of the PCGs was 11,132 bp in size in the mitogenome of *M. magister*, and it contained seven NADH genes, two ATPase genes, and four cytochrome dehydrogenase genes. Nine genes (*cox1–3*, *cob*, *atp8*, *atp6*, *nad2–3*, and *nad6*) were coded in heavy (H) strands, and four genes (*nad1*, *nad4l*, *nad4–*5) were coded in light (L) strands (Figure 1). The 13 typical PCGs began with the standard ATN start codon, which included ATT, ATA, and ATG. Nine PCGs (*cox1–3*, *atp8*, *cob*, *nad4–5*, *nad4l*, *nad2*) were initiated with the standard start codon ATG, three PCGs (*atp6*, *nad1*, *nad3*) started with ATA, and one (nad6) started with ATT. Nine PCGs were predicted to end with TAA, and only two PCGs terminated with the stop codon TAG. Therefore, almost all stop codons in *M. magister* were complete, whereas the *cox2–3* genes appeared to finish with a single T- as an incomplete stop codon. For the termination codons, similar situations have been observed in many crustacean species, and these incomplete stop codons may be completed through post-transitional modification during the mRNA maturation process. 

**Figure 2:**
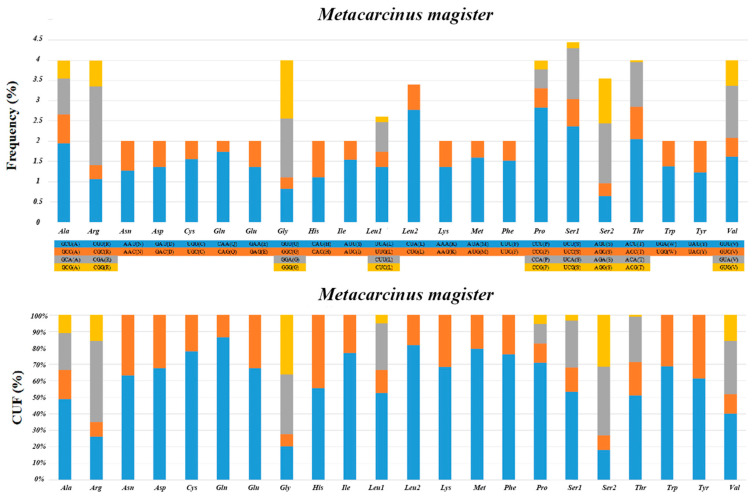
The relative synonymous codon usage (RSCU) analyses and the relative usage frequency (CUF) of the 13 PCGs in *M. magister*.

The relative synonymous codon usage (RSCU) analyses and relative usage frequency (CUF) of the 13 PCGs in *M. magister* are summarized in Figure 2. As in other Brachyura mitogenomes, we found a crucial correlation between codon usage and nucleotide composition. Resembling previous studies in other Brachyuran mitogenomes, the amino acids Ser, Leu, and Pro were the most frequently used, and Leu (UUA) had the largest relative synonymous codon usage. The total number of codons was 3710 in the PCGs of *M. magister,* in addition to the termination codons. The codon families occurred with the same codon usage patterns as in other Brachyuran species, and there was a significant predominance of the usage of one codon containing all of the possibilities in all cases. In *M. magister,* the abundance of the most frequently used codon was equivalent to or less than 50% in the following: Ala, Gly, Ser, and Val. Furthermore, the amino acids that used the most codons in *M. magister* were Ala, Arg, Gly, Leu, Pro, Ser, Thr, and Val. These codons were mostly comprised of A or T nucleotides, which indicated the biased usage of A and T nucleotides in the PCGs of *M. magister*. The overall A + T content of the 13 PCGs was 65.8%, and the AT-skew was negative, which implied a higher occurrence of Ts than As.

### 3.4. Secondary Structure of Transfer and Ribosomal RNAs 

The rRNA genes comprised 2263 bp in *M. magister*. The total AT contents of two rRNAs were 73.2%. The AT-skew and GC-skew in *M. magister* were positive (0.04, 0.14), which indicated that As were slightly more prevalent than Ts and that Gs were much more prevalent than Cs in the tRNA genes. 

*M. magister* contained 22 tRNA genes, the total length of which was 1472 bp. In the tRNAs of *M. magister*, fourteen genes (*trnL2*, *trnK*, *trnG*, *trnR*, *trnN*, *trnS2*, *trnA*, *trnD*, *trnS1*, *trnE*, *trnT*, *trnI*, *trnW*, and *trnM*) were coded on the H-strand, and eight genes (*trnQ*, *trnC*, *trnH*, *trnF*, *trnP*, *trnL1*, *trnV*, and *trnY*) were coded on the L-strand. The lengths of the tRNAs ranged from 64 bp (*trnL2*, *trnG*, *trnF*, and *trnD*) to 73 bp (*trnV*) and showed a strong A + T bias (70.58%). Moreover, the AT-skew and GC-skew in the *M. magister* tRNAs were slightly positive. 

All tRNA genes in *M. magister* displayed a typical cloverleaf secondary structure, except for *trnS1,* which had lost the dihydrouridine (DHU) arm, and the lacking arm was reduced to a small loop (Figure S2). In the postulated cloverleaf structures of Brachyuran tRNAs, the amino acid acceptor arm (AA) contained 7 bp, the DHU arm contained 4 bp, the anti-codon (AC) arm contained 5 bp, and the TΨC contained 5 bp. However, the tRNAs of *M. magister* and *M. nodifrons* had somewhat different and peculiar structures. The variety of tRNA sizes resulted from variations in the lengths of the DHU and TΨC arms; further analysis showed that the TΨC loop for 22 tRNAs within other crabs was inconsistent with the T-loop size in the tRNAs of different species. In addition, truncated tRNAs that lacked the T-loop were also found in other Brachyura species, such as *Somanniathelphusa boyangensis* and *Parasesarma pictum*. Furthermore, a large number of mismatched base pairs were identified in the tRNA genes, including G-U pairs, A-A pairs, and C-U pairs, which particularly existed in the DHU arms (Figure S2); similar mismatches occurred in other Brachyura species.

### 3.5. Main Noncoding Region

Similarly to other Brachyura crabs’ mitogenomes, the different sizes of the mitochondrial genomes between the two species in Cancroidea were mainly due to the variety of sizes of the main noncoding region (mNCR). The mNCR of the *M. magister* mitogenome is currently the longest known mNCR (17,097 bp), with a high A + T content (63.48%), and it was found at a particular location between *trnY* and *trnT*. The nucleotide contents of the 17,097-bp mNCR were 33.77% A, 29.72% T, 9.50% G, and 27.02%. *C. pagurus*, a member of the same family as *M. magister*, also contains an unusual mNCR with a length of 10,987 bp, which is also much longer than that of other crabs (Figure S3).

Microsatellite Tandem Repeats Finder analysis was used, and it was found that TA-rich microsatellites (SSRs) were distributed from position 133 to 16,963. “CT” existed in the A variety of SSRs, and there were “TA” di-nucleotide tandem repeats (Table S3). The mNCR of *M. magister* was AT-rich. The longest “AT” length was 23 bp, and it contained a short “TATA” motif rather than a long string of “TA” motifs. Some relatively short sequences, such as Poly-A, Poly-C, and poly-T stretch, were widely distributed in the mNCR of *M. magister*. 

### 3.6. Phylogenetic Analysis

**Figure 3:**
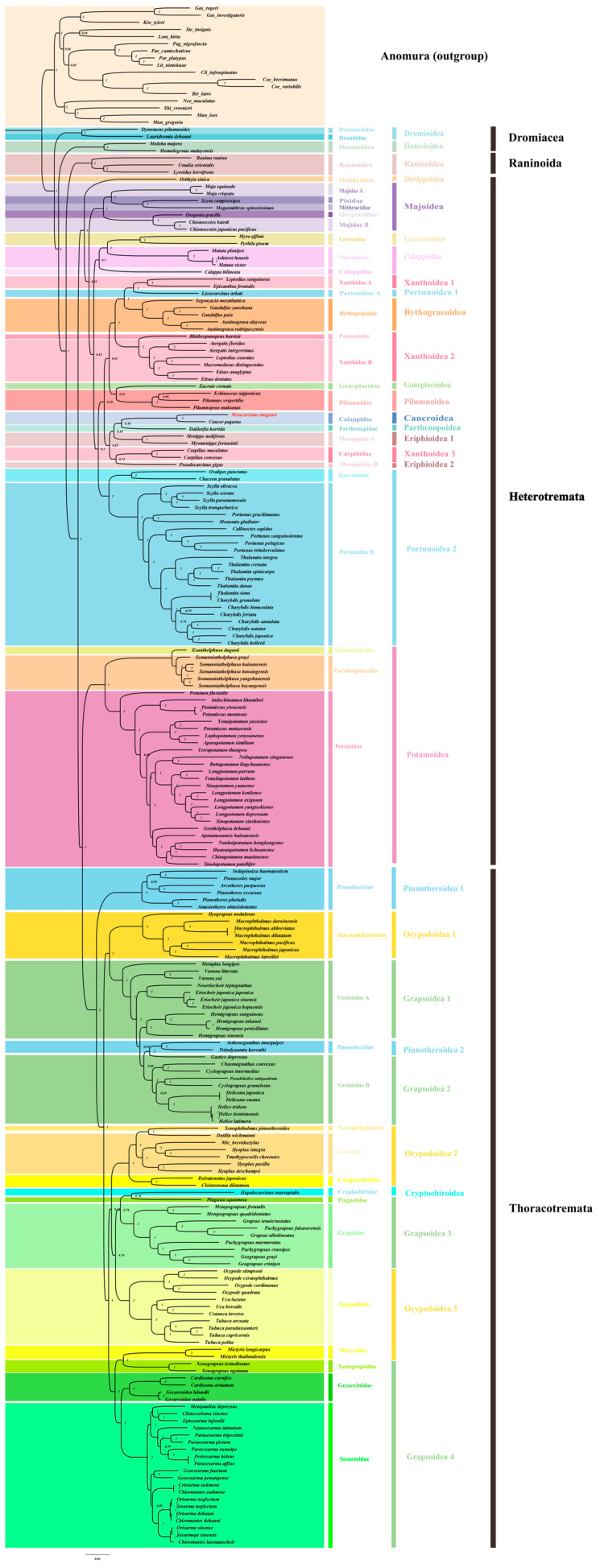
The phylogenetic tree was inferred from the nucleotide sequences of 13 mitogenome PCGs using BI methods, and it included 199 Brachyura species belonging to 39 families and 17 outgroups (Anomura). The dashed lines on the right represent the families and superfamilies of these species.

Based on the complete mitochondrial genome data of Brachyura, we added one Brachyura species, *M. magister* (Decapoda: Brachyura: Cancroidea: Cancridae: Metacarcinus), as a further supplement in order to reconstruct a more comprehensive phylogenetic tree of Brachyura (Figure 3 and Figure S4). A total of 17 sequences from Anomura were used as outgroups in the trees produced with the 13 mitochondrial protein-coding genes from 199 Brachyura species, and the corresponding NCBI accession numbers are shown in Table S2. IQTree in Phylosuite was used for ML analysis, and Mrbayes was used for BI analysis. The phylogenetic relationships resulting from the use of these two methods showed that both trees were largely congruent in their topological structure, except for some shallow branches in Heterotremata (Figure S5). Because of the relatively high bootstrap value, the BI method was used for the subsequent analysis.

As the oldest branches of Brachyura, seven species from Dromiacea and Raninoida were located at the base of this infraorder. In the clade of Eubrachyura, the BI phylogenetic analysis confirmed the paraphyly of Thoracotremata and the monophyly of Heterotremata. 

The two families with the most abundant species in Thoracotremata, Ocypodoidea, and Grapsoidea were shown to have an explicit paraphyletic relationship. The branch topologies from these two superfamilies occurred in an interleaved form on the phylogenetic tree. In addition, the other two groups in Thoracotremata, Pinnotheroidea, and Cryptochiroidea showed monophyletic and paraphyletic relationships, respectively. After the insertion of two Pinnotheroidea species (*Asthenognathus inaequipes*, *Tritodynamia horvathi*), Varunidae was transformed into a paraphyletic group.

Potamoidea and the other Heterotremata species were more closely related to the Thoracotremata species, which led to the paraphyletic topology of Heterotremata. Except for the species of Potamoidea, the other species of Heterotremata emerged with a monophyletic relationship and had the maximum nodal support (BPP/ML = 1/100). At a high level of classification, most Heterotremata superfamilies were found to be monophyletic, except for Xanthoidea and Eriphioidea. It was shown that Xanthoidea was divided into three clades (Xanthoidea1, Xanthoidea2, and Xanthoidea3), and Eriphioidea was divided into two clades (Eriphioidea1 and Eriphioidea2). *M. magister* was classified as Cancroidea, and it was obvious that Cancroidea, Parthenopoidea, and Xanthoidea3 were embedded in Eriphioidea. However, the clades of these superfamilies were not strongly supported in the Bayesian inference (BI) tree or maximum likelihood (ML) tree. Additionally, these clades were clearly different in the results of the two phylogenetic trees (Figure S5).

### 3.7. Gene Rearrangements

The mitochondrial gene orders (MGOs) of *M. magister* (Cancroidea: Cancridae), *C. pagurus* (Cancroidea: Cancridae), and *D. horrida* (Parthenopoidea: Parthenopidae) were observed. *D. horrida* had the same gene order as that of the ancestors of Brachyura. Although the MGOs of *M. magister* were identical to those of *C. pagurus*, they had significant differences from those of the ancestor of Brachyura. In the present study, we discovered the MGO pattern in Cancroidean mitogenomes and preliminarily hypothesized the rearrangement mechanisms. 

**Figure 4:**
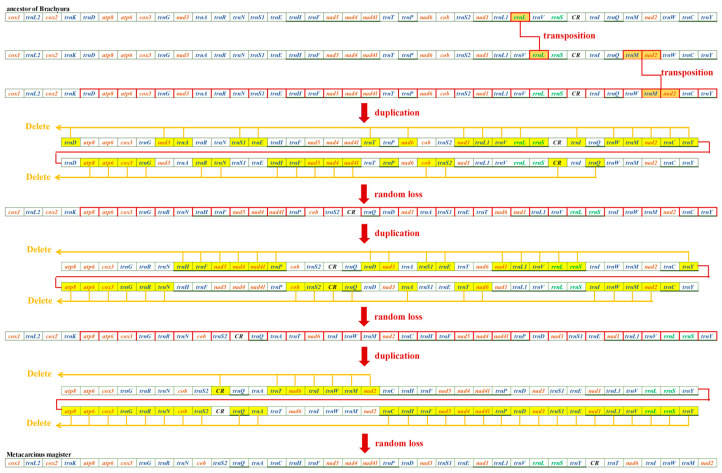
Evolutionary progression from the ancestors of Brachyura to *M. magister*.

Through mechanism analysis, the MGO of Cancroideans required two transposition events and two duplication–random loss (tdrl) events. We found that transpositions occurred first, and this caused the positions of *rrnl* and *trnM*-*nad2* to change. *rrnl* shifted into *trnV* and *rrnS* instead of the usual location between *trnL1* and *trnV*; *trnM*-*nad2* was located between *trnQ* and *trnW*, but here, it was found in *trnW* and *trnC.* These three genes are highlighted in orange (Figure 4). It is widely believed that the two complicated tdrl genes could explain the movement of a large number of mitochondrial genes. All of the mitogenomes involved in the duplication were labeled individually, and all of the random loss genes are highlighted in yellow (Figure 4).

## 4. Discussion

In the present work, the complete mitogenome of *M. magister* has been newly sequenced. Based on supplementary materials, two species comprise the superfamily Cancroidea, namely, *M. magister* and *C. pagurus*. A total of 199 species of Brachyura and 17 outgroup species (Anomuran) were used to explore the taxon’s internal phylogenetic relationships and describe the gene rearrangement patterns in Cancroidea. Two aspects—the particular mitochondrial length of *M. magister* and the genome rearrangement in Cancroidea—must be discussed.

### 4.1. The Particular Mitochondrial Length of M. magister

The reason for the particular length of the mNCR in *M. magister* was assessed from the point of view of mitochondrial length. Rather than differences in gene length, the diversity in mitogenome length was primarily attributed to the existence of IGSs, which included the crucial mNCR [37,38]. The mNCR of the mitochondrial genome was typically the longest IGS region. Because this region contains a number of regulatory elements, including the origin of replication for the heavy strand of the mitochondrial genome, it is termed the mNCR [39]. Moreover, the mNCR contains an abundant A + T content in invertebrates; thus, it is also called an “A + T-rich region”. This region plays a role in the origin of replication and has promoters for the translation of both mitochondrial DNA strands. Therefore, the mNCR of Brachyura provides a promising source for phylogenetic reconstruction based on closely related taxa and polymorphic markers for population genetics [40]. The variations in the IGS length of *M. magister* ranged from 6 bp (*cox2*-*trnK*) to 2593 bp (*trnC*-*trnH*), which might have been caused by the significant size of its mitogenome. For a structure of such an extremely rare length, the 17,097 bp mNCR of *M. magister* was the longest among those reported in Brachyuran species and had a high A + T content (63.48%). This proportion was slightly lower than that in other invertebrate species [41,42], whereas the loop-stem structure was strongly A + T-biased—for example, with A-2 and A-3 (Figure S3). Through an analysis with MITOS, we established that the heavy strand’s origin of replication was contained in the mNCR of *M. magister* and this could certify the hypothesis that the inferred stem-loop structure might be related to the invertebrate heavy-strand or light-strand origin of replication [43].

In the family of Cancridae, *C. pagurus* also has a long noncoding region between its corresponding gene positions. After a comparison, it was found that there was a possible homology in the sequence between *C. pagurus* and *M. magister*. Due to the high mutation rate in the mNCR, such homology is very rare in Brachyura, which may indicate that the two species may have the same origin or similar habitats.

### 4.2. Genome Rearrangement in Cancroidea

The CREx software [33] was used to answer the following question: “How did the MGO of Cancroideans evolve from that of the ancestor of Brachyura?”

Generally, the mitochondrial genome is highly conserved, and gene rearrangement in metazoan mitogenomes is relatively random and rare. Therefore, gene rearrangements can be a tool for researching phylogenetic relationships and they always occur in the mitogenomes of Decapoda. These particular lineages and unusual genomic features, such as this mNCR or the gene order rearrangements in these Menippidae species, could be useful for the reconstruction of evolutionary relationships [42]. Several mechanisms for mitochondrial gene rearrangement have been proposed [44]: transposition [45], duplication–random loss (TDRL), reverse transposition, duplication–nonrandom loss (TDNL), recombination [46], and tRNA mispriming [36]. Combining previous studies with our research, we considered that the TDRL and transposition models were most probably the explanations for the large-scale gene rearrangements that were observed [47]. For some Brachyuran species, mitochondrial gene order (MGO) patterns are shared at the family level, for example in Xanthidae, Orithyiidae, and Grapsidae; however, for others, MGO patterns might be different among species within an identical family or even genus [48]. 

From the perspective of Brachyura gene rearrangements, a hypothesis for the evolution of *M. magister* was proposed as followed. The mNCR of *M. magister* is located between the trnY and trnT genes. In this study, we discovered that two Cancroidea species, one of which was *M. magister,* had the same MGO pattern, but this MGO pattern was different from that of the ancestor of Brachyura. We found that, in Parthenopidae, Daldorfia horrida had the same gene order as that of the ancestor of Brachyura; thus, the rearrangement mechanisms existing in the MGO of Cancroidea were preliminarily predicted with the help of CREx. Figure 4 illustrates a series of gene order rearrangement scenarios that may have led to the MGO in Cancroidea, which underwent TDRL events in addition to transposition. The scope of action of this mechanism is relatively large; thus, the sequence of *M. magister* underwent complicated changes throughout its evolution. We speculated that this long mNCR in *M. magister* was caused by inadequate random loss, which led to the occurrence of pseudogenes and an interval sequence. This speculation was offered by Wang et al. [49]. However, we did not succeed in acquiring gene residuals from the fragment spanning the mNCR in *M. magister*, and this result might indicate peculiar evolutionary mechanisms under high selection pressures. Based on the living environment and diet of *M. magister*, the habitat contained ~53% soft sediments at the bottom, the majority of which were sandy. Additionally, during their period of being omnivorous, larvae also commonly consume heterotrophic prey that ingest toxic algae, and their food is nutritionally deficient [50]. Because of the different living environments and the proliferation of fishing, marine pollution, etc., we speculated that this peculiar mNCR was closely related to these factors. More experiments and analyses were needed to prove this.

Interestingly, it was found that *C. pagurus* had four repetitions of *trnL2*, three repetitions of *trnF*, two repetitions of *trnN,* and two repetitions of *trnY*. By aligning them separately, partial similarity was found in these replicated sequences. Therefore, it is probably true that these tRNA genes were replicated during evolution but were not entirely reserved for other reasons, e.g., changes in the amount of dissolved oxygen or increases in predators. As for the function of extra tRNAs, further research is needed.

## 5. Conclusions

In the present research, the complete mitochondrial genome of *Metacarcinus magister* was analyzed, and the mitogenome contained 13 PCGs, 22 tRNAs, 2 rRNAs, and one mNCR. Unlike the other species of Brachyura, *M. magister* did not have the typical gene order and structure in its mitogenome, and a particular sequence structure occurred in the mNCR. In addition, the rearrangement mechanisms of Cancroidea were discovered. This phenomenon had not been systematically studied in previous research. 

The topological structure of the phylogenetic tree was mostly consistent with that in previous studies of Brachyura, and the researched species, *M. magister,* was located in the shallow branch of Heterotremata. Unlike the gene order of *D. horrida* and the ancestor of Brachyura, a special gene order occurred in the mitochondrial genome of two Cancroidea species. The predicted rearrangement mechanism suggested that two transpositions and two tdrls occurred, and we speculated that these might have had a connection with certain factors, including the living environment, diet, and pollution. We consider the extremely long region and the peculiar rearrangement mechanism important, and these discoveries must be further explored in subsequent research. 

## Figures and Tables

**Table 1:** A summary of the *M. magister* mitogenome.

Gene	Direction	Location	Size	Anticodon	Start Codon	Stop Codon	Intergenic
*cox1*	+	1–1534	1534		ATG	T	−5
*trnL2*	+	1535–1598	64	TAA			8
*cox2*	+	1607–2294	688		ATG	T	0
*trnK*	+	2295–2361	67	TTT			56
*atp8*	+	2418–2576	159		ATG	TAG	−7
*atp6*	+	2570–3244	675		GTG	TAA	−1
*cox3*	+	3244–4034	791		ATG	TA	−1
*trnG*	+	4034–4097	64	TCC			259
*trnR*	+	4357–4422	66	TCG			28
*trnN*	+	4451–4519	69	GTT			849
*cob*	+	5369–6506	1138		ATG	T	0
*trnS2*	+	6507–6573	67	TGA			812
*trnQ*	−	7386–7454	69	TTG			931
*trnA*	+	8386–8450	65	TGC			1799
*trnC*	−	10,250–10,315	66	GCA			2593
*trnH*	−	12,909–12,974	66	GTG			8
*trnF*	−	12,983–13,046	64	GAA			0
*nad5*	−	13,047–14,775	1729		ATG	T	53
*nad4*	−	14,829–16,163	1335		ATG	TAA	−7
*nad4l*	−	16,157–16,453	297		ATG	TAA	6
*trnP*	−	16,460–16,527	68	TGG			2295
*trnD*	+	18,823–18,886	64	GTC			204
*nad3*	+	19,091–19,444	354		ATC	TAA	152
*trnS1*	+	19,597–19,663	67	TCT			0
*trnE*	+	19,664–19,729	66	TTC			1307
*nad1*	−	21,037–21,975	939		GTG	TAA	27
*trnL1*	−	22,003–22,071	69	TAG			47
*trnV*	−	22,119–22,191	73	TAC			1997
*rrnL*	−	24,189–25,584	1396				67
*rrnS*	−	25,652–26,518	867				2012
*trnY*	−	28,531–28,601	71	GTA			0
mNCR	+	28,602–45,698	17,097				0
*trnT*	+	45,699–45,764	66	TGT			25
*nad6*	+	45,790–46,296	507		ATT	TAA	1039
*trnI*	+	47,336–47,402	67	GAT			179
*trnW*	+	47,582–47,649	68	TCA			58
*trnM*	+	47,708–47,773	66	CAT			0
*nad2*	+	47,774–48,790	1017		ATG	TAA	30

**Table 2:** The composition and skewness of the *M. magister* mitogenome.

*M. magister*	T (U) (%)	C (%)	A (%)	G (%)	A + T (%)	AT-Skew	GC-Skew
*Mitogenome*	34.72	22.34	33.97	8.97	68.69	−0.01	−0.43
*PCGs*	39.28	17.26	26.53	16.93	65.81	−0.19	−0.01
*cox1*	36.52	20.92	26.06	16.50	62.57	−0.17	−0.12
*cox2*	35.78	20.23	30.06	13.93	65.84	−0.09	−0.18
*atp8*	43.40	19.50	28.93	8.18	72.33	−0.20	−0.41
*atp6*	38.69	21.43	27.08	12.80	65.77	−0.18	−0.25
*cox3*	36.11	22.66	25.35	15.88	61.46	−0.18	−0.18
*cob*	38.27	21.47	25.97	14.29	64.24	−0.19	−0.20
*nad5*	39.08	10.36	27.83	22.74	66.90	−0.17	0.37
*nad4*	42.25	9.74	26.74	21.27	68.99	−0.22	0.37
*nad4l*	42.42	8.75	25.59	23.23	68.01	−0.25	0.45
*nad3*	38.38	19.89	28.29	13.45	66.67	−0.15	−0.19
*nad1*	42.74	10.47	24.15	22.65	66.88	−0.28	0.37
*nad6*	41.52	23.78	25.93	8.77	67.45	−0.23	−0.46
*nad2*	40.71	23.50	25.37	10.42	66.08	−0.23	−0.39
*tRNAs*	34.04	12.64	36.55	16.78	70.58	0.04	0.14
*rRNAs*	34.42	9.72	38.75	17.10	73.18	0.06	0.28
mNCR	29.72	27.02	33.77	9.50	63.48	0.06	−0.48

## Data Availability

The data that support this study are available from the NCBI and are provided in Table S1.

## Supplementary Materials

The following supporting information can be downloaded at: https://www.mdpi.com/article/10.3390/genes15040437/s1, Figure S1: The image of *M. magister*; Figure S2: Secondary structure diagram of 22 tRNAs; Figure S3: The Secondary structure diagram of *M. magister* mNCR; Figure S4: The ML phylogenetic tree of Brachyura; Figure S5: A comparison of topology of Heterotremata in BI and ML; Table S1: Sequences of universal primers; Table S2: The GenBank accession numbers of 132 species in this study; Table S3: The Microsatellite Tandem Repeats in *M. magister*.
