# Supplementary figures and images for: Analysis of the Overlength Main Noncoding Region in Metacarcinus magister (Decapoda: Brachyura) and a Phylogenetic Study of the Cancroidea Species
Source: Genes (Basel). 2024 Mar 29;15(4):437. doi: 10.3390/genes15040437 (PMC11049931; doi:10.3390/genes15040437)

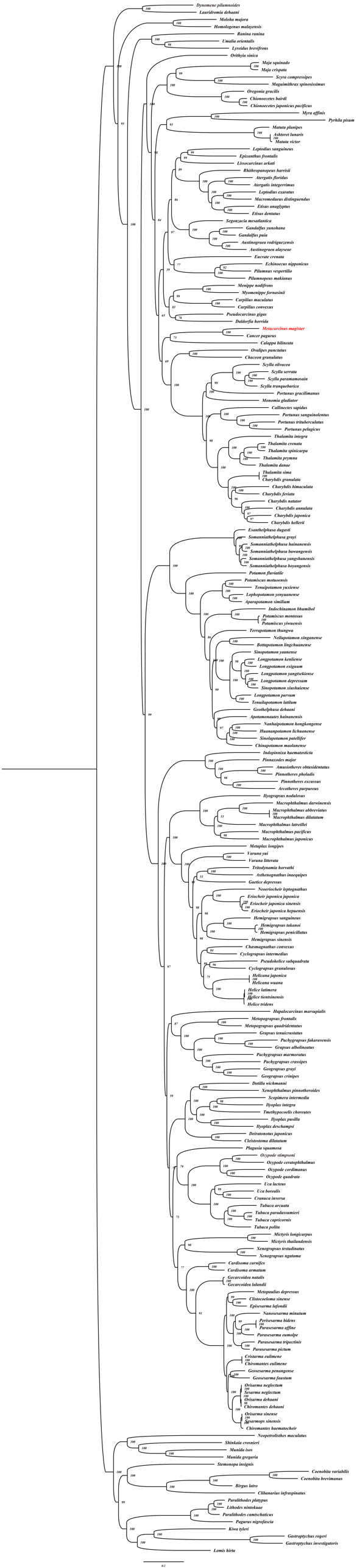

Supplement: Supplementary file 1 [file genes-15-00437-s001.zip › Figure S4.pdf]

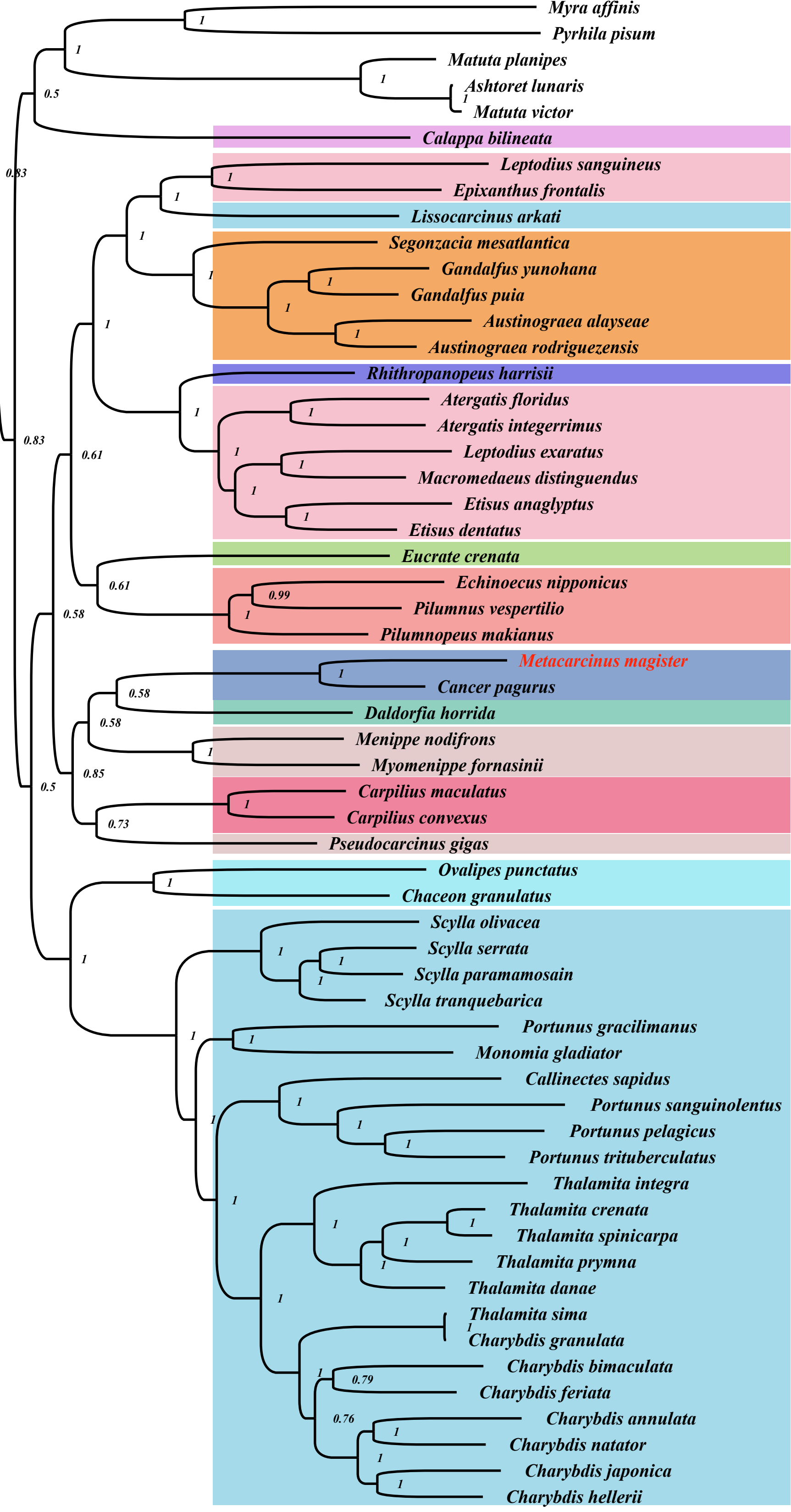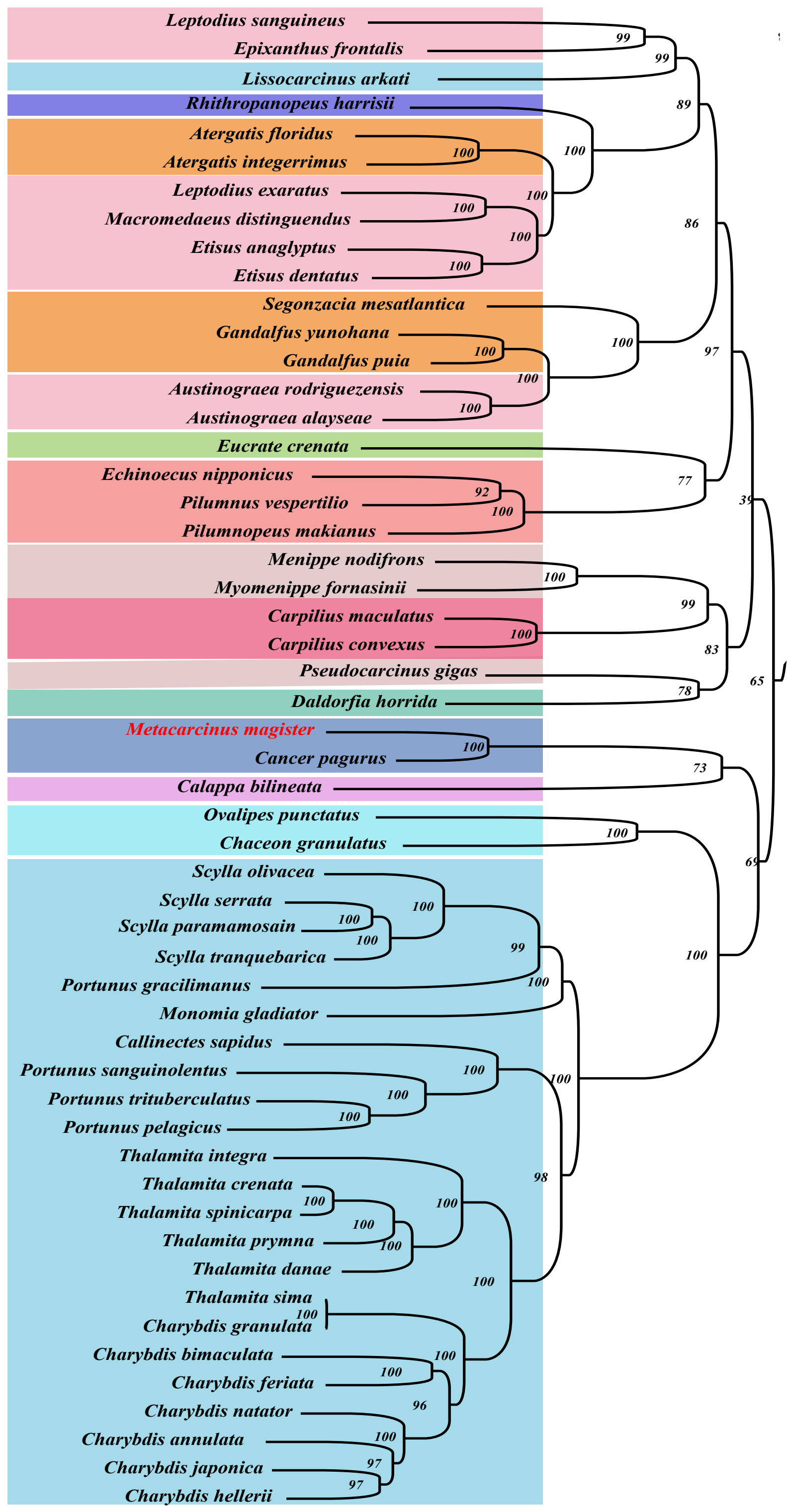

Supplement: Supplementary file 1 [file genes-15-00437-s001.zip › Figure S5.pdf]

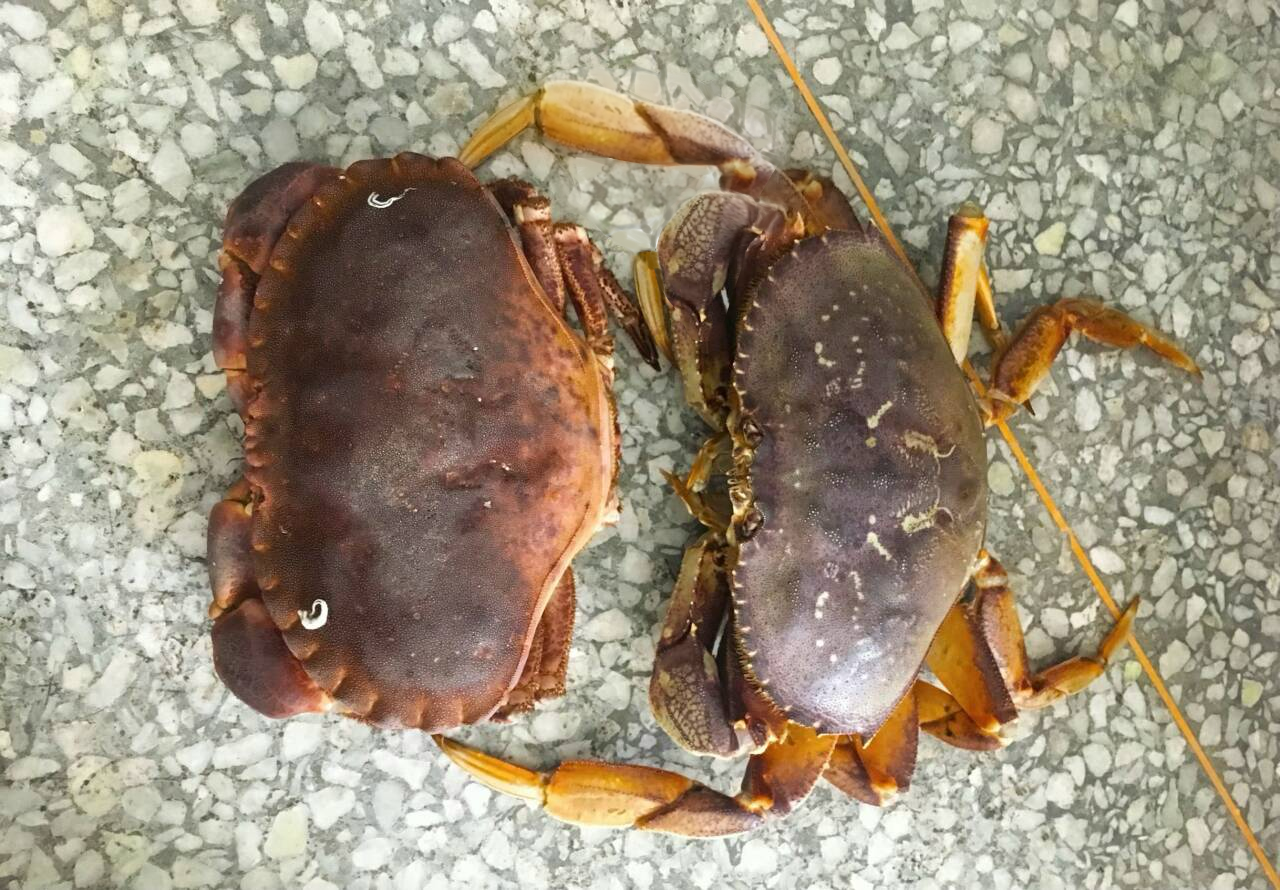

Supplement: Supplementary file 1 [file genes-15-00437-s001.zip › Figure S1.png]

## A: Metacarcinus magister

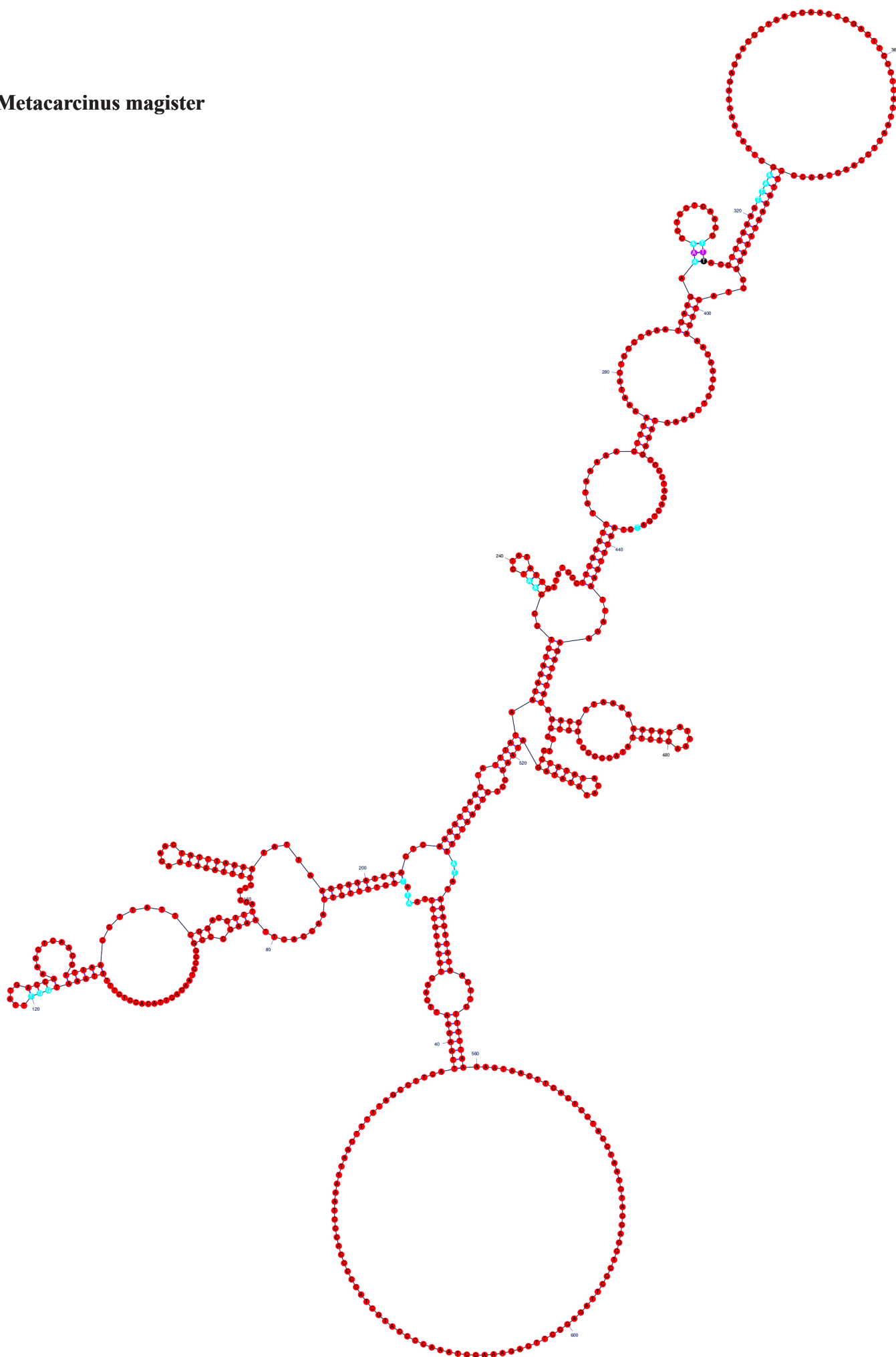

$dG = -3.42$  CR

Supplement: Supplementary file 1 [file genes-15-00437-s001.zip › Figure S3.pdf]
