# Supplementary material for: Analysis of the Overlength Main Noncoding Region in Metacarcinus magister (Decapoda: Brachyura) and a Phylogenetic Study of the Cancroidea Species
Source: Genes (Basel). 2024 Mar 29;15(4):437. doi: 10.3390/genes15040437 (PMC11049931; doi:10.3390/genes15040437)
Supplement: Supplementary file 1 [file Figure S2.pdf › Figure S2.pdf]

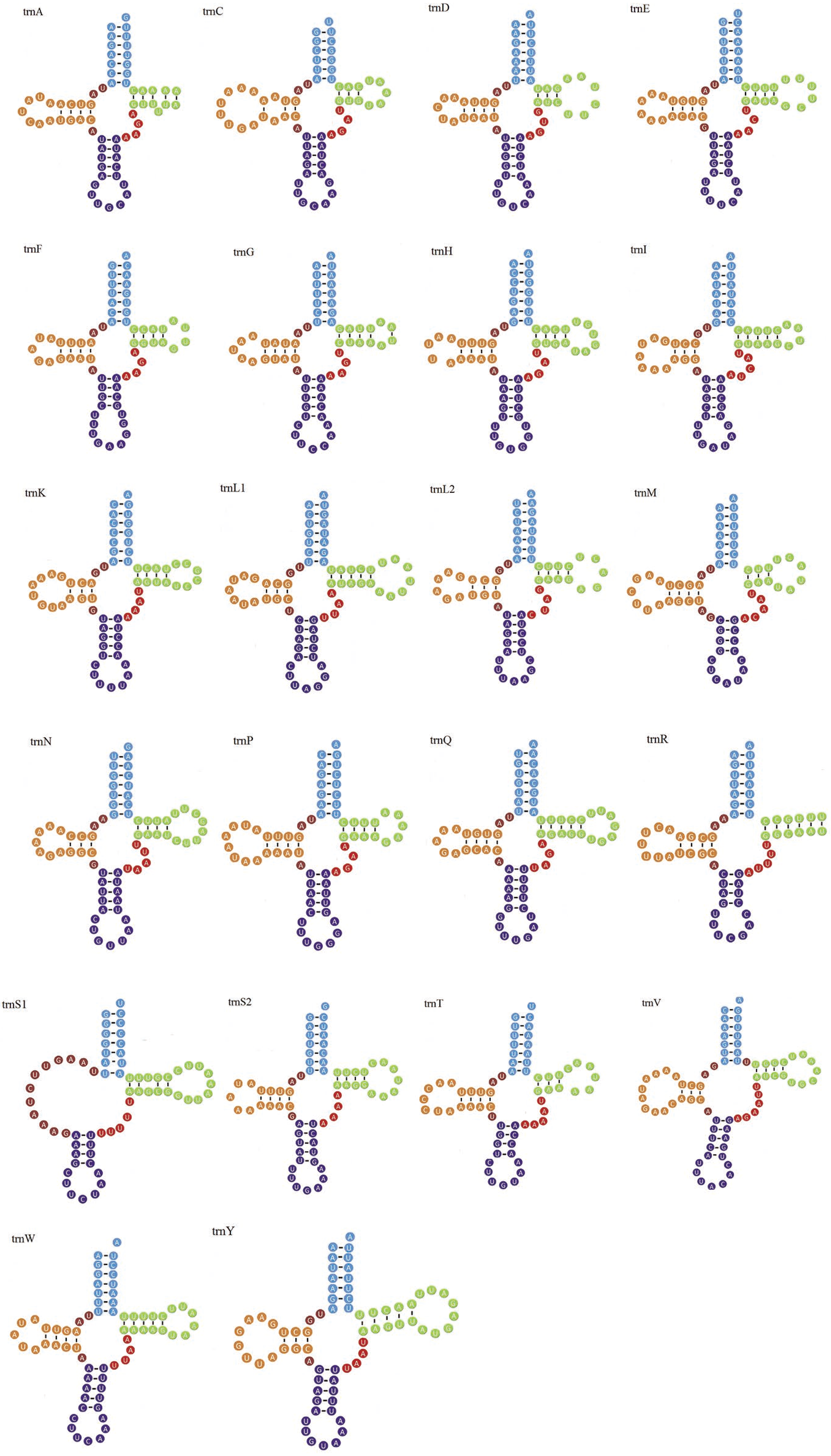
trnA
trnC
trnD
trnE
trnF
trnG
trnH
trnI
trnK
trnL1
trnL2
trnM
trnN
trnP
trnQ
trnR
trnS1
trnS2
trnT
trnV
trnW
trnY
